# Cryptic speciation in a benthic isopod from Patagonian and Falkland Island waters and the impact of glaciations on its population structure

**DOI:** 10.1186/1742-9994-5-19

**Published:** 2008-12-19

**Authors:** Florian Leese, Anna Kop, Johann-Wolfgang Wägele, Christoph Held

**Affiliations:** 1 Alfred Wegener Institute for Polar and Marine Research, Marine Animal Ecology, PO Box 12 0161, D-27515 Bremerhaven, Germany; 2 Ruhr University Bochum, Department of Animal Ecology, Evolution and Biodiversity, Universitaetsstrasse 150, D-44801 Bochum, Germany; 3 York University, 4700 Keele St., Toronto M3J 1P3, Canada; 4 Zoologisches Forschungsmuseum Alexander Koenig, Adenauerallee 160, D-53113 Bonn, Germany

## Abstract

**Background:**

The Falkland Islands and Patagonia are traditionally assigned to the Magellan Biogeographic Province. Most marine species in Falkland waters are also reported from southern Patagonia. It remains unclear if relatively immobile, marine benthic, shallow-water species maintain gene flow, and by what mechanism. Recurrent fluctuations in sea level during glacial cycles are regarded as a possible mechanism that might have allowed genetic exchange between the regions. However, the realized genetic exchange between the Falkland Islands and Patagonia has never been estimated.

**Results:**

This study analyses the genetic structure of three populations of the marine shallow-water isopod *Serolis paradoxa *(Fabricius, 1775) from the Falkland Islands and southern Patagonia (central Strait of Magellan and the Atlantic opening) applying seven nuclear microsatellites and a fragment of the mitochondrial 16S rRNA gene. Both marker systems report highest genetic diversity for the population from the central Strait of Magellan and lowest for the Falkland Islands. The estimated effective population sizes were large for all populations studied. Significant differentiation was observed among all three populations. The magnitude of differentiation between Patagonia and the Falkland Islands (16S: uncorrected p-distance 2.1%; microsatellites: standardized F'_ST _> 0.86) was an order of magnitude higher than between populations from within Patagonia. This indicates that there is currently no effective gene flow for nominal *S. paradoxa *between these two regions and it has been absent for time exceeding the last glacial maximum. We argue that specimens from the Strait of Magellan and the Falkland Islands very likely represent two distinct species that separated in the mid-Pleistocene (about 1 MY BP).

**Conclusion:**

The results of this study indicate limited gene flow between distant populations of the brooding isopod *Serolis paradoxa*. The patterns of genetic diversity suggest that the only recently inundated Strait of Magellan was colonized by different source populations, most likely from Atlantic and Pacific coastal waters. Our results demonstrate that more systematic testing of shared faunal inventory and realized genetic exchange between Patagonia and the Falkland Islands is needed before a consensus concerning the position of the Falkland Islands relative to the Magellan zoogeographic province can be reached.

## Background

**Figure 1:**
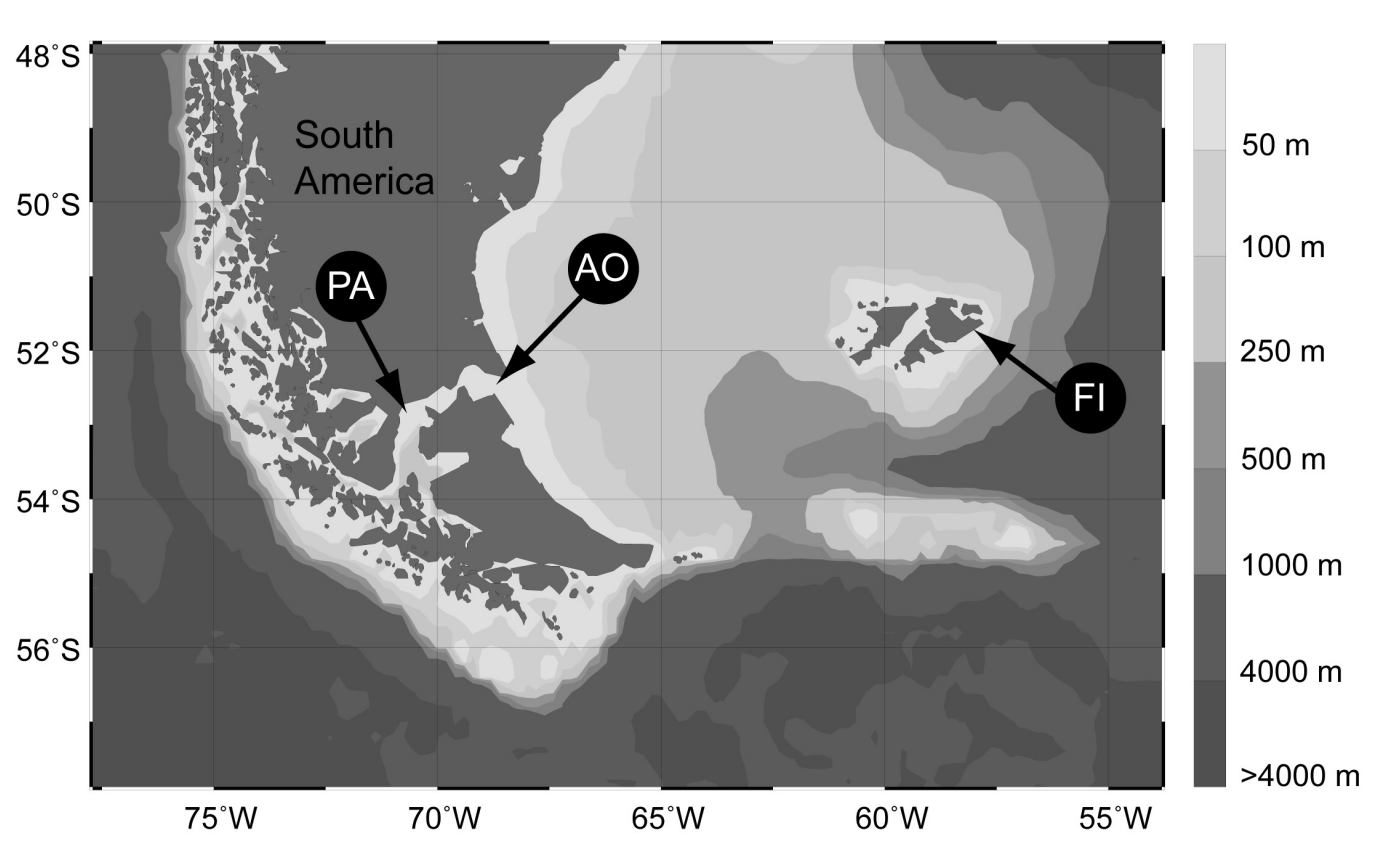
**Sampling sites of *Serolis paradoxa *in the Strait of Magellan near Punta Arenas (PA), the opening to the Atlantic Ocean (AO) and the Falkland Islands (FI)**.

Present-day distribution of a species is the result of a complex interplay between (1) extrinsic factors such as isolation of landmasses, climatic conditions and availability of niches and (2) intrinsic factors such as dispersal capability and physiological tolerance. Extrinsic factors typically influence the distribution of many species in the same way because they act on an ecosystem scale. Over time, this leads to a characteristic assemblage of species with similar distribution patterns within larger geographical areas, so called biogeographic provinces, and distinct gaps between them [1]. In the Southern Hemisphere, the Magellan Biogeographic Province has obvious close ties to the whole of South America to which it is connected today; some of its species inventory, however, stems from times before the Gondwana breakup [2,3]. The Falkland Islands are connected to the South American shelf and located approximately 500 km to the east of Patagonia (Figure 1). Based on the current knowledge of their species inventory, the Falkland Islands are commonly assigned to the Magellan Biogeographic Province [4-7], sometimes as a more distinct 'subregion' of this province ([8] and references therein). The geologic history of the Falkland Islands is completely detached from continental South America as the islands drifted to their current position on a microplate that originally formed part of the African continental plate. Their current position was reached approximately 130 MY BP [9-11]. Close biogeographic ties between the Falkland Islands and Patagonia are widely accepted and even more plausible in the marine realm because here biotic exchange does not depend on the existence of land bridges [12-15]. Furthermore, major ocean current systems facilitate dispersal of specimens. This is generally supported by elevated levels of gene flow among the few species investigated so far in this region [16-19].

In this context, the relatively few reports of species endemic to the Falkland Islands are not unexpected [8,20-22]. On the whole, evidence from marine species supports that the Falkland Islands form a part of the Atlantic Magellan Biogeographic Province and that migration of species between the continental South America and the Falkland Island is occurring repeatedly. However, recent molecular studies have shown that unrecognized cryptic species may be more common than previously assumed [15,17,23-33]. They indicate that morphological and ecological similarity may be an unreliable piece of evidence on which to base taxonomic identifications and, by extension, the definition of biogeographic provinces derived from them.

In this study we investigate spatial partitioning of intraspecific molecular polymorphisms in nominal *Serolis paradoxa *(Fabricius, 1775), a marine benthic shallow-water isopod, using two independent genetic marker systems. *S. paradoxa *was originally described from the Falkland Islands but is also frequently reported from the Strait of Magellan, the Patagonian coastal shelf (Atlantic and Pacific side), and also from regions further to the equator [5,20]. For the current taxonomic status and synonyms of *S. paradoxa *see [3]. The vertical distribution of *S. paradoxa *ranges from shallow waters (about 5 m, Held pers. observ., Lopaz-Gappa pers. observ.) down to 113 m [34]. Although in the Magellan region *S. paradoxa *can be locally very abundant [35] it is often encountered at medium densities (about 1 ind/m^2^[36], Mutschke and Rios pers. comm.). Like almost all isopods, *S. paradoxa *lacks free-swimming distribution stages and broods its offspring in a ventral brood pouch, the marsupium, and is thus expected to be limited in its dispersal capabilities. No information on the life cycle and the duration of *S. paradoxa *are known. Based on significantly extended embryonic stages of Antarctic serolid isopods in comparison to non-Antarctic isopods [37] it can be assumed that embryonic development, maturation and brooding of *S. paradoxa *from the cool-temperate regions, each stage lasts several months. Altogether, *S. paradoxa *is expected to have very limited dispersal capacity due to these factors.

Direct measurements of dispersal and migration over large geographical distances provide a poor means of assessing effective gene flow. The small number of immigrants needed per generation to appreciably change the genetic structure of a population will not be picked up by realistic sampling schemes. Indirect genetic estimates use tools that interpret the genetic structure of a population as a result of past genetic influx and thus represent an easier and more reliable method [38]. However, in this context historic extrinsic factors that may have exerted a structuring force must be considered when estimating present-day population structure. One extrinsic factor known to have had a major impact on genetic structure and distribution of species are glaciation events [39]. Their influence on the marine fauna is two-fold: large-scale glaciations may directly render entire coastal habitats unavailable during glacial maxima [40-42] and also lead to a decrease in sea level of up to 130 m [41]. The latter may disrupt inshore habitats on either side of an emerging barrier (e.g. appearance of the Panama land bridge [12], or connect shallow water habitats that are disjunct during periods of high sea level (additional file 1).

The focus of the present study is the genetic structure of *S. paradoxa *from the Falkland Islands and the Patagonian shelf. The present-day situation suggests that the deeper waters on the South American shelf may present an insurmountable barrier to *S. paradoxa *inhabiting shallow waters around Patagonia and the Falkland Islands. Historically, the lower sea level during glacial maxima may have connected both regions and facilitated migration between habitats thus counteracting independent lineage sorting in the two regions.

By investigating the coherence between gene pools and construction of an approximate timeline, we test whether the disruptive or unifying forces predominated and if the influence of the last glaciations exerted a major influence on the evolutionary history of *S. paradoxa*. We also test whether the major age difference between marine habitats in the central Strait of Magellan and around the Falkland Islands (< 14 KY BP [43-50] and millions of years [51], respectively) exerted a measurable influence on the genetics of populations living there today. In particular, we test for differences in genetic diversity and patterns of recent population expansions or secondary contact of colonizers from the Atlantic and the Pacific side in central Magellan Strait. We hypothesize that populations in the central Strait of Magellan are genetically less diverse than populations from the coast or the Falkland Islands due to recent range expansion into the Magellan Strait subsequent to the retreat of the glaciers after the last glacial maximum (LGM).

## Materials and methods

### Taxon sampling

Specimens from the Falkland Islands (FI) were collected by dredging in shallow waters (< 20 m) near Port Stanley from a Zodiac during the ICEFISH 2004 expedition. Specimens from the Strait of Magellan near Punta Arenas (PA) were collected in January 1997 by CH SCUBA diving at two neighbouring stations (500 m apart) and specimens from the Atlantic opening of the Strait of Magellan (AO) were provided from the 2^nd ^Cruce Estrecho in 2003, by Carlos Rios and Erika Mutschke, Universidad de Magallanes, Punta Arenas (see Figure 1 and Table 1). Animals were immediately preserved in 96% ethanol. Microsatellite analyses were performed for 35 specimens from PA, 33 from AO and 23 from FI. A subset of 27 specimens from PA, 22 from AO and 22 from FI were analysed for variation of the 3'-terminus of the mitochondrial 16S rRNA gene.

**Table 1:** Sampling sites (PA = Strait of Magellan near Punta Arenas, AO = Atlantic opening of the Strait of Magellan, FI = Falkland Islands).

**Region**	**Depth [m]**	**Year**	**Cruise**	**N_MSAT_**	**N_mtDNA_**
Strait of Magellan near Punta Arenas	8 m	1996	SCUBA diving (CH)	35	27
Opening of the Strait of Magellan to the Atlantic Ocean	<20 m	2003	2nd Cruce Estrecho	33	22
West Falkland Islands	15 m	2004	ICEFISH 2004	23	22

Numbers of specimens studied using nuclear microsatellite markers (N_Msat _and mitochondrial markers (N_mtDNA_) of the populations of *S. paradoxa *investigated in this study.

### DNA extraction, PCR, sequencing/genotyping

Total DNA was extracted from muscle tissue using the Qiagen DNeasy Mini Kit according to the standard tissue protocol. Only 100 μl of elution buffer were used to increase DNA concentration.

#### Microsatellites

Microsatellite markers Spa04, Spa12, Spa34, Spa35, Spa39, Spa42 and Spa43 [32] were applied to assess intraspecific genetic polymorphisms for all specimens from the three sampling sites. Standard 15 μl reactions consisted of 1× PCR HotMaster Buffer, 0.2 mM dNTPs, 0.5–0.75 μM of each primer (one labelled, one unlabelled), 0.03 U/μl HotMaster *Taq *(Eppendorf, 5-Prime), 0.5 M Betaine (Sigma Aldrich) and 5–20 ng of genomic DNA. Cycling conditions on an epgradient thermocycler (Eppendorf) were 2 min at 94°C followed by 30 to 34 cycles with 20 s at 94°C, 15 s at annealing temperature, 30 s at 65°C. A final extension step of 45 minutes at 65°C was performed to reduce *in vitro *artefacts due to incomplete adenylation of products [see [32] for details]. PCR products were visualized on 2% TBE agarose gels, diluted 1–15 fold with molgrade water (CARL ROTH) and 1 μl of the diluted product was denatured in a mixture of 14.7 μl HI-DI formamide with 0.3 μl GeneScan ROX 500 size standard (both Applied Biosystems). Allele length scoring was performed using the software GENEMAPPER 4.0 (Applied Biosystems). To minimize genotyping errors [52,53], up to four independent reactions were performed on a subset of samples to estimate allele calling errors.

#### 16S rDNA

The universal primers 16Sar and 16Sbr [54] were used for amplification. Reactions were carried out in 25 μl volumes with 1× HotMaster reaction buffer, 0.2 mM dNTPs, 0.5 μM of each primer, 0.025 U/μl HotMaster *Taq *(Eppendorf, 5-Prime). Reaction conditions were: Initial denaturation for 2 min at 94°C followed by 36 cycles of 20 s at 94°, 15 s at 46°C and 80 s at 65°C plus a final elongation step of 5 min at 65°C. PCR products were purified using Qiagen QIAquick or Eppendorf Perfectprep Gel cleanup kits. Cycle-sequencing was performed in 10 μl reaction volumes using 1 μM of either 16Sar or 16Sbr primer, 1 μl of the purified template DNA and the BigDye Terminator Kit 3.1 chemistry (Applied Biosystems) according to the recommendations of the manufacturer. Reactions were purified according to the 'modified protocol' of the Qiagen DyeEx Kit. Sequencing was conducted on an ABI 3130xl sequencer.

### Data analysis

#### Microsatellites

Raw data were checked and corrected for genotyping errors using the software MICRO-CHECKER version 2.2.3 [55] and DROPOUT version 1.3 [56]. In addition, MICRO-CHECKER was used to test for the presence of null alleles in populations, i.e. alleles that fail to amplify due to substitutions in the primer binding regions. Corrected genotype tables were converted to specific software formats using the software MSTOOLKIT version 3.1 [57] and CONVERT version 1.3.1 [58]. The program ANIMALFARM version 1.0 [59] was used to test for loci with significantly disproportionate variances that may bias allele-size based distance estimates such as Slatkin's or Rousset's R_ST _estimates [60,61]. Tests for Hardy Weinberg equilibrium (HWE) and linkage disequilibrium (LD) were performed using GENEPOP version 4.0.6 [62]. Parameter settings: 10,000 dememorization steps, 50 batches, 20,000 MCMC sampling steps. HWE tests aim at testing whether there is a statistically significant deviation of genotype frequencies from those expected according to Mendelian inheritance. Linkage disequilibrium occurs when two genomic loci are not inherited independently, e.g. due to physical linkage or other processes at population level hindering independent recombination of loci.

To assess partitioning of genetic variability within individuals, subpopulations and regions, we performed hierarchical analyses of molecular variance (AMOVA) using ARLEQUIN version 3.11 [63]. Therefore, populations PA and AO were assigned to one group, while FI constituted the other group. In addition, single and multilocus inbreeding coefficients (F_IS_) and pairwise population coancestry coefficients (F_ST_, similar to Weir and Cockerham's Theta) were estimated as in [64] using GENEPOP. We also calculated pairwise allele-size based differentiation estimates, R_ST_, according to [61] using GENEPOP. Significance was assessed by exact G tests as implemented in GENEPOP. The interpretation of the F_ST _values from multiallelic data is problematic because their maximum values depend on the amount of within-population variation and even in the absence of any shared allele often fail to reach the theoretical maximum of 1 [65-67]. We therefore applied a standardization approach suggested by Hedrick [67] for calculations of G_ST _and derived by Meirmans [68] for Analysis of Variance frameworks (ANOVA).

The main principle of this standardization approach is to correct the maximum possible value for F_ST _as follows: F_ST(max) _is calculated using GENEPOP applying the sampling bias correction suggested by Meirmans [68] using the Software RECODEDATA[68]. F'_ST _was subsequently calculated by dividing F_ST _by this inferred maximum value.

The standardized F'_ST _measure calculated range from 0 (populations equifrequent for all alleles) to 1 (populations fixed for different alleles) and therefore makes interpretation of the degree of subdivision much easier and facilitates comparing results among studies.

In addition to these ANOVA based coancestry estimates we performed individual assignment tests using the program STRUCTURE, version 2.2.2 [69] to investigate population subdivision. The advantage of the Bayesian clustering algorithm of STRUCTURE is that no classification of populations has to be done *a priori*. Assuming HWE and no or only weak LD within subpopulations, STRUCTURE assigns individual genotypes probabilistically to populations and calculates the likelihood of the genotype dataset for a given number of populations (K), i.e. ln Pr (D|K) for K = 1 to K = n, using a Markov Chain Monte Carlo algorithm [69,70]. For the *S. paradoxa *data set, the most likely number of populations was inferred without making assumptions on geographic origin of individuals. The number of MCMC steps needed to reach convergence was first estimated by comparing run lengths between 10,000 and 2,000,000 steps. Convergence was generally reached with <5,000 steps. Therefore, for the parameter sets 10 independent runs with a burn-in of 5,000 and subsequent 100,000 MCMC steps were performed with and without assuming recent admixture in the prior model, and considering alleles as correlated and uncorrelated. The number of clusters (K) to infer was defined from K = 1 to K = 4 to allow detection of potential cryptic subpopulations. Alpha was inferred from the data for each population separately. Results from 10 independent runs were analysed in CLUMPP, version 1.1.1 [71] to compute a consensus membership coefficient Q-matrix from all 10 independent Q-matrices. Both the individual Q-matrix and averaged population Q-matrix were visualized using DISTRUCT, version 1.1 [72].

To assess estimates of the present effective population size (*N*_*e*_), we applied the linkage disequilibrium method proposed by Hill (1981) [73], modified by Waples [74] to account for a bias correction when sample size is much smaller than effective population size. This method is implemented in the program LDNE, version 1.3 [75]. Calculations of *N*_*e *_and the confidence intervals (CI) were estimated considering alleles with a frequency of c ≥ 0.05 and c ≥ 0.02 and ≥ 0.01, respectively.

Tests for historical population bottlenecks were performed using the program BOTTLENECK[76]. Tests implemented in this program are based on the hypothesis that populations that have experienced recent reductions in their effective population size (*N*_*e*_) show a reduction in both allelic richness and heterozygosity. In populations decreasing in size, the number of alleles (*N*_*A*_) drops faster than heterozygosity [77] and therefore the observed heterozygosity is larger than the expected heterozygosity (*H*_*O *_> *H*_*E*_). Conversely, in expanding populations often the number of alleles increases faster than heterozygosity until equilibrium is reached. From the comparison of both parameters, allelic diversity and heterozygosity, it is possible to make inferences regarding historical demography of a population. For each locus and population BOTTLENECK computes distribution of *H*_*E *_expected from the observed *N*_*A*_, given the sample size (n) under the assumption of mutation-drift equilibrium. This distribution is obtained through simulating the coalescent process of n genes under the three possible mutation models, i.e. a) the Infinite Allele Model (IAM), b) the Two-Phase Model (TPM), c) the Stepwise-Mutation Model (SMM). As recommended by Cornuet and Luikart [78] we tested several proportions of the SMM for the TPM (70–90%). Statistical significance of the parameters were inferred applying a Sign-test and a Wilcoxon-rank-test [76,78,79].

#### 16S rDNA

Assembly of forward and reverse strands and editing was performed using the software SEQMAN (Dnastar, Lasergene) and GENEIOUS version 4.0.2 (Biomatters Ltd.). Sequence alignment was performed using MUSCLE version 3.6 [80]. The alignment required no manual correction based on secondary structure information [81]. Sequence variation was analyzed using MEGA 4.0 [82]. Gene diversity and nucleotide diversity according to Nei [83] and Theta based on the number of segregating sites, Theta (S), were calculated with ARLEQUIN version 3.11. Genetic differentiation between populations and between regions [(PA + AO) vs. FI] were assessed using an F_ST _and AMOVA framework as implemented in ARLEQUIN. Assuming neutrality, evidence of a population expansion was tested applying Tajima's *D *[84] and Fu's *F*_*S *_statistic [85] as implemented in ARLEQUIN applying a coalescent simulation approach generating 10,000 selectively neutral samples for assessment of significance of results. A test for sudden population expansion based on the pairwise mismatch distribution was calculated using ARLEQUIN and significance was assessed by 50,000 pseudo replicates.

A statistical parsimony network with a 95% connection-probability limit was created for the 490-bp alignment using TCS version 1.21 [86]. In addition, two outgroup sequences of the serolid isopods *Cuspidoserolis luethjei *and *Cuspidoserolis johnstoni *(GenBank accession numbers AJ269802, AJ269803; see [24]) were aligned to the *S. paradoxa *sequences using MUSCLE, resulting in a 492-bp alignment. This alignment was used to calculate a neighbor joining tree [87] with bootstrap support (1000 replicates) based on uncorrected p-distances using PAUP* version 4b10 [88].

The coalescent-based MCMC approach implemented in the software BEAST[89] was used to date the splitting event between the different *Serolis paradoxa *lineages applying both a strict and a relaxed molecular clock model. The sequence model HKY85 was used for modelling sequence evolution [90] together with a predefined mutation rate of 0.37% per million years [91], based on a molecular clock for the serolid isopod *Ceratoserolis trilobitoides*. Dating times and confidence intervals (CI) were filtered using TRACER version 1.4 [92].

## Results

### Microsatellites

Seven microsatellite loci were analysed for three populations. All loci were highly polymorphic for all three populations (Table 2). The number of alleles per microsatellite locus ranged from 6 to 23. The observed heterozygosity ranged from 0.0 (Spa39, all specimens homozygous for populations FI) to 0.886. Total heterozygosity was highest in PA, lower in AO and lowest in FI (Table 2). Significant deviations from HWE were detected for loci Spa04 and Spa39 (Table 2). Analyses with MICRO-CHECKER indicate that null alleles may be the cause for inflated homozygosity for these loci. No significant global LD was observed after sequential Bonferroni correction [93]. Based on allele frequencies, the geographically intermediate population AO is clearly more similar to PA than FI (additional file 2). The allele distribution of all microsatellite loci reveal strongly differing frequency spectra with several private and almost fixed different allele patterns between regions (e.g. loci Spa04, Spa12, Spa35, Spa43). Allele length spectra differ between populations but overlap (additional file 2).

**Table 2:** Total number of specimens scored for each locus (*N_S_*), number of different alleles (*N_A_*), inbreeding coefficient (*F_IS_*), observed heterozygosity (*H_O_*) and expected heterozygosity (*H_E_*) for the seven microsatellites and three populations of *Serolis paradoxa*.

	PA	AO	FI	Mean *N*_*A*_/locus
Spa04				
*N*_*S*_	32	33	23	
*N*_*A*_	5	5	4	4.667
*F*_*IS*_	0.316**	-0.075	0.539*	
*H*_*O*_	0.769	0.485	0.130	
*H*_*E*_	0.682	0.452	0.279	
Spa12				
*N*_*S*_	35	33	23	
*N*_*A*_	6	4	3	4.333
*F*_*IS*_	0.172	-0.028	-0.012	
*H*_*O*_	0.257	0.121	0.087	
*H*_*E*_	0.310	0.118	0.086	
Spa34				
*N*_*S*_	35	33	23	
*N*_*A*_	20	12	13	15.000
*F*_*IS*_	0.040	0.102	0.206	
*H*_*O*_	0.886	0.667	0.696	
*H*_*E*_	0.922	0.741	0.872	
Spa35				
*N*_*S*_	34	30	17	
*N*_*A*_	7	9	5	7.000
*F*_*IS*_	-0.015	0.138	0.286	
*H*_*O*_	0.676	0.633	0.353	
*H*_*E*_	0.667	0.733	0.490	
Spa39				
*N*_*S*_	35	27	13	
*N*_*A*_	17	16	6	13.000
*F*_*IS*_	0.322**	0.299**	1.000**	
*H*_*O*_	0.629	0.630	0.000	
*H*_*E*_	0.923	0.893	0.788	
Spa42				
*N*_*S*_	35	33	23	
*N*_*A*_	14	14	11	13.000
*F*_*IS*_	0.034	0.119	0.069	
*H*_*O*_	0.886	0.788	0.826	
*H*_*E*_	0.907	0.892	0.886	
Spa43				
*N*_*S*_	35	33	22	
*N*_*A*_	2	4	5	3.667
*F*_*IS*_	-0.033	0.230	-0.085	
*H*_*O*_	0.40	0.364	0.636	
*H*_*E*_	0.388	0.470	0.588	

Mean *N*_*A *_per location	10.14	9.14	6.71	8.667
Mean *H*_*O*_	0.600	0.527	0.390	
Mean *H*_*E*_	0.686	0.614	0.570	
*F*_*IS*_	0.110**	0.094**	0.181**	

*/** refer to markers that depart from HWE at P < 0.05 and P < 0.01, respectively.

Results of ANIMALFARM confirmed that none of the loci contributed disproportionally to distance-based differentiation estimates after Bonferroni or Sidak adjustment of the significance level.

Analyses of the AMOVA indicate that most variation is distributed among individuals (Table 3). Φ_IS _and the global multilocus inbreeding coefficient F_IS _are significantly positive for all populations (Table 2, Table 3) indicating further within-population structure. However, a large proportion of variation is distributed among the major geographical regions [(PA + AO) vs. FI] and only a minor but nonetheless significant proportion between populations (Table 3). Strong differences between the two regions were observed for allele frequency patterns at all loci. In particular loci Spa04, Spa12 and Spa43 are nearly fixed for different alleles in populations from the two major regions [(PA, AO) vs. FI] (additional file 2) whereas PA and AO reveal very similar allele frequency patterns. The pronounced differences separating FI from Patagonia are expressed by the high and significant pairwise F_ST _and R_ST _estimates between population PA and FI and populations AO and FI (Table 4). Differentiation estimates were even higher between populations AO and FI. In contrast, F_ST _estimates among populations PA and AO were low albeit significant (P = 0.0005). R_ST _estimates were lower than F_ST _estimates in this study and did not support significance differentiation between PA and AO (Table 4). In general, R_ST _is hypothesized to be larger if an appreciable amount of differentiation between populations is not only caused by drift but by independent mutations in the different, isolated populations according to a stepwise mutation model (SMM). Consequently, R_ST _distance measures are considered a 'memory' of past mutations [60]. F_ST _is superior to R_ST _when populations have diverged mostly by means of random genetic drift and migration m (i.e. mutation rate << migration).

**Table 3:** Hierarchical analyses of molecular variance (AMOVA) among *Serolis paradoxa *populations within and between two regions using 7 microsatellite markers.

**Component of differentiation**	**df**	**variation [%]**	**Φ statistics**	**P**
Among regions	1	30.99	Φ_CT _= 0.310	0.329
Among populations within regions	1	1.64	Φ_SC _= -0.024	0
Among individuals within populations	88	8.02	Φ_IS _= 0.119	0
Within individuals	91	59.35	Φ_IT _= 0.407	0

(2 populations from the Strait of Magellan, one population from the Falkland Islands)

**Table 4:** Genetic differentiation among populations of *Serolis paradoxa *from three stations as assessed by F-statistics (F_ST_, lower diagonal) and R-statistics (R_ST_, upper diagonal), based on seven polymorphic microsatellite loci.

	**PA**	**AO**	**FI**
**PA**	-	-0.006	0.217*
**AO**	0.023* (0.065*)	-	0.258*
**FI**	0.322* (0.863*)	0.376* (0.901*)	-

* refers to P < 0.001 (exact G test). F_ST _values in parentheses are differentiation estimates standardized according to Meirmans (2006).

Standardized pairwise F_ST _calculates [67] in this study showed very strong pairwise population differentiation between Patagonia and the Falklands (PA vs FI: 0.86; AO vs FI: 0.91), and much smaller values among the Magellan Strait populations (PA vs AO: 0.063). These values demonstrate that both regions are almost fixed for different alleles at the seven loci investigated. When removing locus Spa39, which is biased due to the presence of null-alleles in population FI, the standardized values did not change, however, the non-standardized F_ST _values were almost twice as high (data not shown).

**Figure 2:**
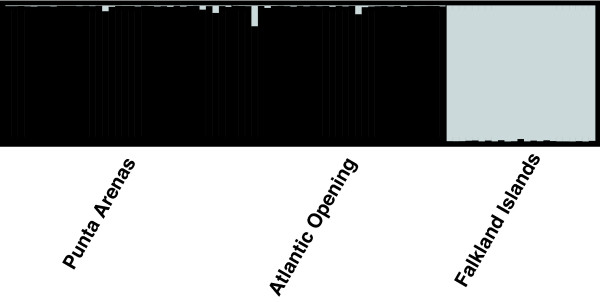
**Results of cluster analyses performed with STRUCTURE (admixture model, allele frequencies correlated) with the highest log likelihood probability**. The graphs display the consensus membership coefficients matrices (Q-matrices) for 91 individuals from three populations of *Serolis paradoxa *using seven microsatellite loci. The genotype of each individual is represented by a single bar, where the proportion of the colour refers to the probability of assignment to a certain cluster.

Inferring the most likely number of populations without making assumptions concerning their delimitation, STRUCTURE identified only two very distinct clusters, which correspond to the two major regions (AP+AO vs. FI; Figure 2). When using the no-admixture model, all individuals are correctly assigned to the two regions with admixture proportions of 1.000 and 0.000, respectively. There was no additional substructure within populations (PA, AO, FI), i.e., when analysing the populations separately Ln Pr (D|K) was highest for K = 1. No significant differentiation between population PA and AO was detected (compare F_ST _estimates, Table 4).

Estimating the present effective population sizes using the LD approach [74], we consistently received negative *Ne *estimates with confidence intervals ranging from negative values to infinity, thus indicating very large population sizes [75]. When testing for recent demographic contractions or expansions by looking for deviations from mutation-drift equilibrium under different mutation models using BOTTLENECK we found a significant heterozygosity deficiency under particular mutation models. For population AO there was a significant heterozygosity deficit under both SMM and TPM models (additional file 3), which provides strong evidence for recent population expansion. For FI the evidence for recent population expansion was somewhat weaker: a significant heterozygosity deficit was detected only using the SMM and the Wilcoxon test (P = 0.0195, additional file 3). Thus results of BOTTLENECK do not provide evidence for a similarly drastic decline and subsequent recovery in population size for PA. For population AO under a TPM and a strict SMM, the significant excess of heterozygosity may indicate that this population is expanding presently. Although the evolutionary dynamics of microsatellites are not fully understood [94,95] it is commonly accepted that the IAM model is not an appropriate descriptor of the mutational dynamics of microsatellite markers and hence that its application often leads to unrealistic conclusions.

In summary, the results from the microsatellite analyses provide evidence for moderate differentiation between the two Patagonian populations and very strong subdivision between populations from Patagonia and the Falkland Islands. Genetic diversity was highest in the center of the Strait of Magellan, lower near its opening towards the Atlantic Ocean and lowest around the Falkland Islands. All populations showed a significant heterozygosity deficit corroborated by high F_IS _values (Table 2) which may be indicative for inbreeding of local populations.

### 16S rDNA

**Figure 3:**
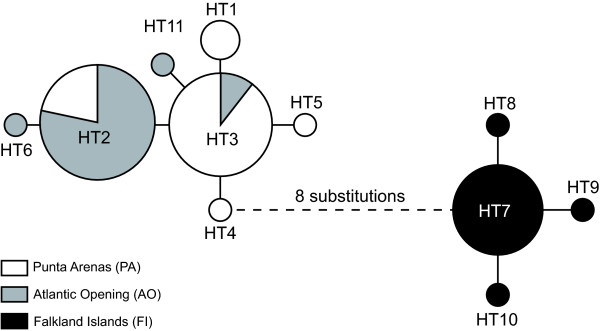
**Statistical parsimony network of 16S rDNA haplotypes of *Serolis paradoxa* from the Strait of Magellan (white and grey) and the Falkland Islands (black)**. Branches in subnetwork represent one substitution except for the branch connecting HT4 and HT7, which differ by eight mutations.

**Figure 4:**
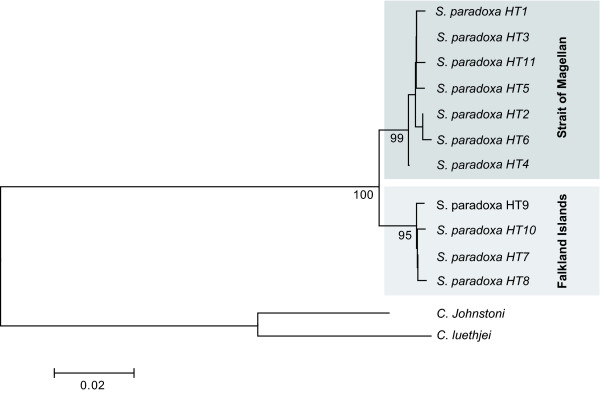
**Neighbor joining tree based on uncorrected p-distances of 3'-terminus of the mitochondrial 16S rRNA gene for the 11 haplotypes from *S. paradoxa *sampled from Patagonia (HT1-HT6, HT11) and the Falkland Islands (HT7-10)**. Sequences of *Cuspidoserolis luethjei*, AJ269802 and *C. johnstoni*, AJ269803 [15] were used as outgroup. Numbers on branches represent bootstrap support (1000 replicates).

We sequenced a 490 bp fragment of the 16S rRNA gene for a subset of 27 specimens from population PA, 22 from AO and 22 from FI (Table 1) to test whether the strong pattern of differentiation inferred using fast evolving microsatellites is also traceable with a slower evolving gene. The amplified fragment was AT-rich as typical for this gene (A 39.3%, C 13.1%, G 14.0%, T 33.6%; [54]). Of the seventeen polymorphic positions, ten were parsimony informative and seven represented singletons. Substitutions were located only in loop regions of the rRNA gene fragment (folding model: *Drosophila melanogaster *16S rRNA, [81]). Eleven haplotypes were characterized (HT1-HT11, Table 5). The statistical parsimony network constructed is characterized by two shallow subnetworks (≤ 4 segregating sites) representing the Patagonian vs. the Falkland Islands populations, which are connected by a long internal branch (8 segregating sites, Figure 3). None of the haplotypes was shared among specimens from different regions. Phylogenetic analyses revealed that specimens from both regions form two reciprocally monophyletic clades each supported by high bootstrap values (Figure 4). The average uncorrected pairwise distance between both groups was 2.1% (1.7% for transitions; 0.4% for transversions). Variation within groups was an order of magnitude lower (0.3% among FI and 0.4% among PA and AO; Figure 4). These values are amongst the lower values observed between reproductively isolated species of serolid isopods and other crustaceans [24,26-28,31]. This dominant partitioning of genetic diversity by regions was supported by high and highly significant (P < 0.001) values for pairwise population differentiation (F_ST_) between populations from both regions: PA:FI F_ST _= 0.95; AO:FI, F_ST _= 0.97). The results of the AMOVA indicate that 90.48% of the total variance is partitioned between the two regions [(PA and AO) vs. (FI)], corresponding to a high value of Φ_CT _= 0.905 (Table 6). However, a lower but highly significant proportion of total variance was also distributed between populations AO and PA (4.70%), corresponding to the value of Φ_SC _of -0.494. Pairwise differentiation estimates support that gene flow is also restricted within the Strait of Magellan (PA:AO F_ST _= 0.45; P < 0.001).

**Table 5:** Distribution of the n = 71 16S rDNA sequences on the two sampling locations and GenBank accession numbers.

**Haplotype**	**PA**	**AO**	**FI**	**Accession no**.
HT1	3	0	0	EU419766
HT2	5	18	0	EU419767
HT3	17	2	0	EU419768
HT4	1	0	0	EU419769
HT5	1	0	0	EU419770
HT6	0	1	0	EU419771
HT7	0	0	19	EU419772
HT8	0	0	1	EU419773
HT9	0	0	1	EU419774
HT10	0	0	1	EU419775
HT11	0	1	0	FJ457033

**Table 6:** Hierarchical analyses of molecular variance (AMOVA) among *Serolis paradoxa *populations within and between two regions based on the 16S rDNA data.

**Component of differentiation**	**df**	**variation [%]**	**Φ statistics**	**P**
Among regions	1	90.48	Φ_CT _= 0.905	0.333
Among populations within regions	1	4.70	Φ_SC _= -0.494	0
Within populations	68	4.82	Φ_ST _= 0.952	0

(2 populations from the Strait of Magellan, one population from the Falkland Islands)

Nucleotide diversity and gene diversity were highest for population PA, lower for AO and lowest for FI (Table 7). Estimates of Theta (S) were also higher for PA than for AO and FI (Table 7). Tajima's *D *and Fu's *Fs *were negative and significantly different from zero for FI, and for Fu's *Fs *only for population AO (Table 7). Recent population expansions are frequently associated with negative values of *D *and *Fs *because under these circumstances mutation generates more and closely related haplotypes than are eliminated by genetic drift. It should be considered that according to Fu [85] a significance level of 5% corresponds to P = 0.02, thus Fu's *Fs *for population AO is not significant. The mismatch analyses could not the reject the assumptions of sudden population expansion for all populations (additional file 4). In summary, there is a stronger signature for population expansion in AO and FI than in PA based on the 16S rDNA data.

**Table 7:** Genetic diversity and neutrality indices for the 16S rDNA data sets.

**parameter**	**Magellan Strait (PA)**	**Atlantic Opening (AO)**	**Falkland Islands (FI)**
nucleotide diversity	0.0014 ± 0.0012	0.0009 0.0010	0.0006 ± 0.0008
haplotype diversity	0.5755 ± 0.0952	0.3333 0.1243	0.2600 ± 0.1202
Theta (S)	1.038 ± 0.5900	0.8230 0.5237	0.823 ± 0.5237
Tajima's *D*	-0.945 (P = 0.200)	-1.240 (P = 0.085)	-1.729 **(P = 0.009**)
Fu's *Fs*	-1.819 (P = 0.059)	-1.827 **(P = 0.034)**	-2.889 (**P = 0.001**)

Dating of the time to the most recent common ancestor (tMRCA) between both *S. paradoxa *lineages using a strict molecular clock for the mutation rate differed for the two monophyletic lineages. For the Patagonian taxa as ingroup the tMRCA inferred was 0.948 MY (5% CI 0.344 MY, 95% CI 1.658 MY) and for the Falkland Islands lineage 0.643 MY (5% CI 0.136 MY, 95% CI 1.207 MY). Thus, from both inferences, evidence for a splitting event in the mid-Pleistocene is supported.

## Discussion

The genetic variability within the nominal species *Serolis paradoxa *turned out to have extensive spatial structure. The differences in mutation rates and coalescent dynamics of the two marker systems help describe present-day population structure and reconstruct historical demographic processes.

### Two genetically distinct lineages

There is strong evidence of divergence between populations from Patagonia and the Falkland Islands, supported by microsatellite and mitochondrial data. The dominant feature of the intraspecific variability of mitochondrial DNA data for the Patagonian populations (PA and AO) and the Falkland Island population is that populations form two shallow subnetworks, corresponding to the two geographic regions. The nuclear microsatellite markers support the geographic partitioning of variation with high and significant F_ST_, R_ST _differentiation values and strong support from Bayesian cluster analyses (Figure 2).

The geographic positions of our sampling locations along an East-West axis might suggest testing for isolation by distance effects (IBD). However, in the context of this study, the IBD is an inappropriate method and it is unlikely that this would become more meaningful even if more intermediate sampling locations were available. This is because the central Strait of Magellan became available for (re)colonization only very recently, approximately 9–14 KY BP [46,48]. This rapid range expansion is typically accompanied by loss of alleles and an excess of homozygosity [96] which violates a mutation-drift equilibrium assumed by the IBD model. Investigating distance effects on the distribution of intraspecific variance inside the Magellan Strait offers a means to trace the recolonization of this young habitat and would be appropriate for IBD but this requires more fine-scaled sampling and is outside the scope of this paper.

Absence of effective gene flow between the Falkland Islands and Patagonia is strongly suggested by nearly fixed population specific differences in fast evolving microsatellites and the perfect congruence of haplotype identity and geography for the 16S rDNA data. The long branch connecting the two groups of haplotypes (Figure 3) and their reciprocal monophyly (Figure 4) indicates complete lineage sorting in both groups. The magnitude of genetic differentiation between 16S genotypes is on the order of magnitude known for reproductively isolated species [26-28,97]. Speciation ultimately involves the irreversible disruption of a once contiguous gene pool into two [98]. The recognition of species thus centers around direct or indirect evidence for gene flow between them. Our data from two independent molecular markers are in line with the expectations of two independently evolving lineages. The patterns and magnitude of the remaining differences do not suggest the presence of additional cryptic species inside (PA and AO) vs. (FI), they indicate, however, that gene flow is restricted even within the Strait of Magellan. The congruence between both marker systems supports that the 16S rRNA gene tree reflects the species tree rather than being a result of shared ancestral polymorphisms [99] or other processes affecting mitochondrial genes (see [100] for review).

### Evolutionary history of nominal *Serolis paradoxa*

Southern Hemisphere glaciations differently affected both regions: The Falkland Islands were little affected by glacial advances [51], thus *S. paradoxa *was able to survive by following the rising and falling sea levels. In Patagonia, however, major parts of today's distribution of nominal *S. paradoxa *became unavailable due to ice coverage and/or low sea levels. Western Patagonia was covered by a contiguous ice shield similar to the Antarctic Peninsula today and the central Strait of Magellan was inundated only after the LGM, approximately 14-9 KY BP [46]. Contrary to the situation around the Falkland Islands where *S. paradoxa *was presumably continuously present over evolutionary times, this species was forced to immigrate into the Strait of Magellan only recently after the retreat of the glaciers. Surprisingly, genetic diversity estimates for population PA from central Magellan Strait (Table 7) indicate that the population has the highest genetic diversity and shows almost no signs for recent population expansion (Table 7, additional file 3, 4), although colonization of a new habitat is often accompanied by a loss of genetic diversity (founder event). In comparison, population FI is less diverse for the 16S rDNA with one dominant haplotype only (HT7) and reveals strong evidence for recent population expansion. Diversity estimates decline from population PA in the west to population FI in the east, which seems counterintuitive as the effects of past glaciations are likely to have been much more severe for PA than for AO and FI. This apparent contradiction may, however, be explained by the fact that the Magellan Strait was recolonized after the LGM not only from the Atlantic but also the Pacific side, thus receiving allelic diversity from different source populations. In the contact zone in the central Magellan Strait, this scenario explains the inflated genetic diversity estimates for PA.

In summary, our data are in agreement with the following scenario: Populations of an ancestral species were separated geographically and evolved in allopatry (Falkland Islands vs. Pacific and Atlantic side of Patagonia). Applying a rate for the accumulation of substitutions in 16S rDNA estimated by Held [91] for the serolid isopod *Ceratoserolis trilobitoides *(Eights, 1833) with a rate of 0.37% per MY for transitions and transversions, the time of divergence was estimated to have occurred several hundreds of thousands of years before present. Thus the initial separation of lineages predates the last glaciations and took place in mid-Pleistocene (average estimates 0.643 – 0.948 BP). In view of the strong genomic signatures of differentiation between Patagonia and the Falkland Islands we must therefore reject the hypothesis that low sea levels during glacial periods led to significantly elevated levels of gene flow between populations of *S. paradoxa *due to greater proximity of shallow-water habitats. A similar argument applies to potential migration between Patagonia and the Falkland Islands via passive rafting on drifting substrates. Although there are major directional ocean currents that frequently transport substrates suitable for transportation of even rather immobile species [101-103] this apparently played no role in the recent evolutionary history of *S. paradoxa*. This species exclusively inhabits soft-bottom shallow waters and is frequently half-buried in the sediment (Held pers. observ.). Its capability to colonize new island habitats and maintain genetic continuity across barriers to dispersal and over evolutionary times is therefore small. Further sampling effort should focus on sampling specimens from the West Falkland Islands. It cannot be excluded that members of both lineages live in sympatry today.

### Reliability and systematic bias in differentiation estimates

The equilibrium F_ST _estimate for totally isolated populations based on microsatellites can reach the maximum value of F_ST _= 1 only theoretically. Due to the high mutation rate of microsatellites [94,104,105] and often a restricted allelic spectrum ([106], but see [107]), the intrapopulation variability is generally very high in particular after a long time of independent evolution of large populations. Applying Meirman's standardization approach for pairwise F_ST_, differentiation between PA and FI is 0.86, between AO and FI 0.91 and among the Magellan Strait populations PA and AO 0.063 and thus about three times larger than without this correction. These values underpin that populations from Patagonia and the Falkland Islands are almost fixed for different alleles at the seven loci investigated. The results point out the importance of the recently introduced standardization approach [67,68] in order to allow for easier comparison and interpretation of the data. Differentiation estimates of the 16S rDNA yield comparable results. Differentiation was significant between all three populations. Although populations AO and PA shared the most common haplotypes, F_ST _estimates between PA and AO revealed much higher differentiation than inferred using microsatellite data. The most plausible explanation is that the fourfold smaller effective population size of mitochondrial DNA [108] lead to much stronger effects of genetic drift, resulting in higher differentiation estimates.

In principle, differentiation estimates can also be biased due to comparing samples obtained in different years (PA: 1997, AO: 2003, FI: 2004). However, as only few years, corresponding to even fewer generations of *S. paradoxa*, separate the samples and no major disturbances in the regions were reported for the time in-between the samplings. Thus, we regard this a negligible issue.

Concerning the dating of the split between the two lineages, it must be stated that genetic distances between two lineages increase much faster than predicted by molecular clocks if populations experience population bottlenecks [65]. Thus, the realistic tMRCA between the two lineages might be shorter than the estimated mean using the molecular clock. In addition, it is not entirely certain if the molecular clock can be applied to *S. paradoxa*. The time estimates are based on 16S rRNA substitution rates commonly used for other Crustacea [97].

### Taxonomic and conservation status of the newly delimited species

The genetic data strongly suggest that nominal *Serolis paradoxa *(Fabricius, 1775) consists of two reproductively isolated species one of which occurs in Patagonia while the other is presumably confined to shallow waters around the Falkland Islands. As the type was originally described by Fabricius as *Oniscus paradoxum *Fabricius, 1775 from the Falkland Island the species from Patagonia is in need of formal description and a scientific name.

The occurrence of cryptic species has important implications for the conservation of biodiversity in general [109]. If a cryptic species is not recognized, unique and endangered local faunas cannot be efficiently protected. However, the estimates of effective population size for both species contained inside nominal *Serolis paradoxa *imply that both are highly abundant and neither needs to be considered endangered.

## Conclusion

In summary, our data prove low but significant differentiation among populations within the Strait of Magellan and the absence of effective gene flow among populations from the Strait of Magellan and the Falkland Islands. In fact, specimens from both regions belong to two cryptic lineages that probably diverged in the mid-Pleistocene and may already represent reproductively isolated species. The 16S rDNA data supports a genetically rich central Strait of Magellan population, an intermediate population near the Atlantic opening of the Strait of Magellan and a genetically depauperate Falkland Island population. The results are in line with the expectations of colonization of the central Strait of Magellan from both sides of Patagonia after the last glacial maximum approximately 9-14 KY after deglaciation of the habitat and rise of sea levels.

While the fauna of the Falkland Islands has often been accepted to share most of their faunal inventory with Patagonia our results indicate that shallow water species with low mobility may in fact be strongly differentiated populations of one species or even reproductively isolated species.

## Competing interests

The authors declare that they have no competing interests.

## Authors' contributions

CH and FL designed the study and sampled the specimens. FL and AK carried out the molecular genetic analyses. FL performed the computational analyses, was responsible for the interpretation of population genetic data and drafted the manuscript. CH served as PI for the project and made substantial contributions to the interpretation of the data and together with FL wrote the final manuscript. JWW participated in the coordination of the study and contributed to revising the manuscript. AK helped draft the manuscript. All authors read and approved the final version of the manuscript.

## Supplementary Material

Additional file 1 **Terrestrial (black) and marine habitats (grey, 50 m bathymetry isolines) available at last glacial maximum (approximately 21 KY BP, left panel) and at the present interglacial (right panel)**. During last glacial maximum the sea level was considerably lower thus bringing shallow water habitats of the Patagonian Coast and the Falkland Islands in close proximity. Adapted from [49]. Click here for file

Additional file 2 **Allele frequencies at seven microsatellite loci for three *Serolis paradoxa *populations (PA = Punta Arenas, AO = Atlantic opening of the Strait of Magellan, FI = Falkland Islands)**. Private alleles are shown in bold. Click here for file

Additional file 3 **Statistical tests for significant heterozygosity (H) excess or deficiency in three populations of *Serolis paradoxa *(PA, AO, FI) assuming three different mutation models (IAM, TPM, SMM)**. P-values of the Sign Test and Standardized Differences Test and one-tailed probability for heterozygosity deficiency are based on a 1000 permutations. Significant P-values are printed in bold. The TPM was adjusted to allow for 80% mutations according to a SMM and 20% to an IAM model. Click here for file

Additional file 4 Mismatch analysis based on the 16S rDNA data sets. Confidence intervals (CI give the 5% and 95% values, respectively for the parameters estimated by 50,000 bootstrap replicates. Click here for file
