# Supplementary figures and images for: Cryptic speciation in a benthic isopod from Patagonian and Falkland Island waters and the impact of glaciations on its population structure
Source: Front Zool. 2008 Dec 19;5:19. doi: 10.1186/1742-9994-5-19 (PMC2644686; doi:10.1186/1742-9994-5-19)

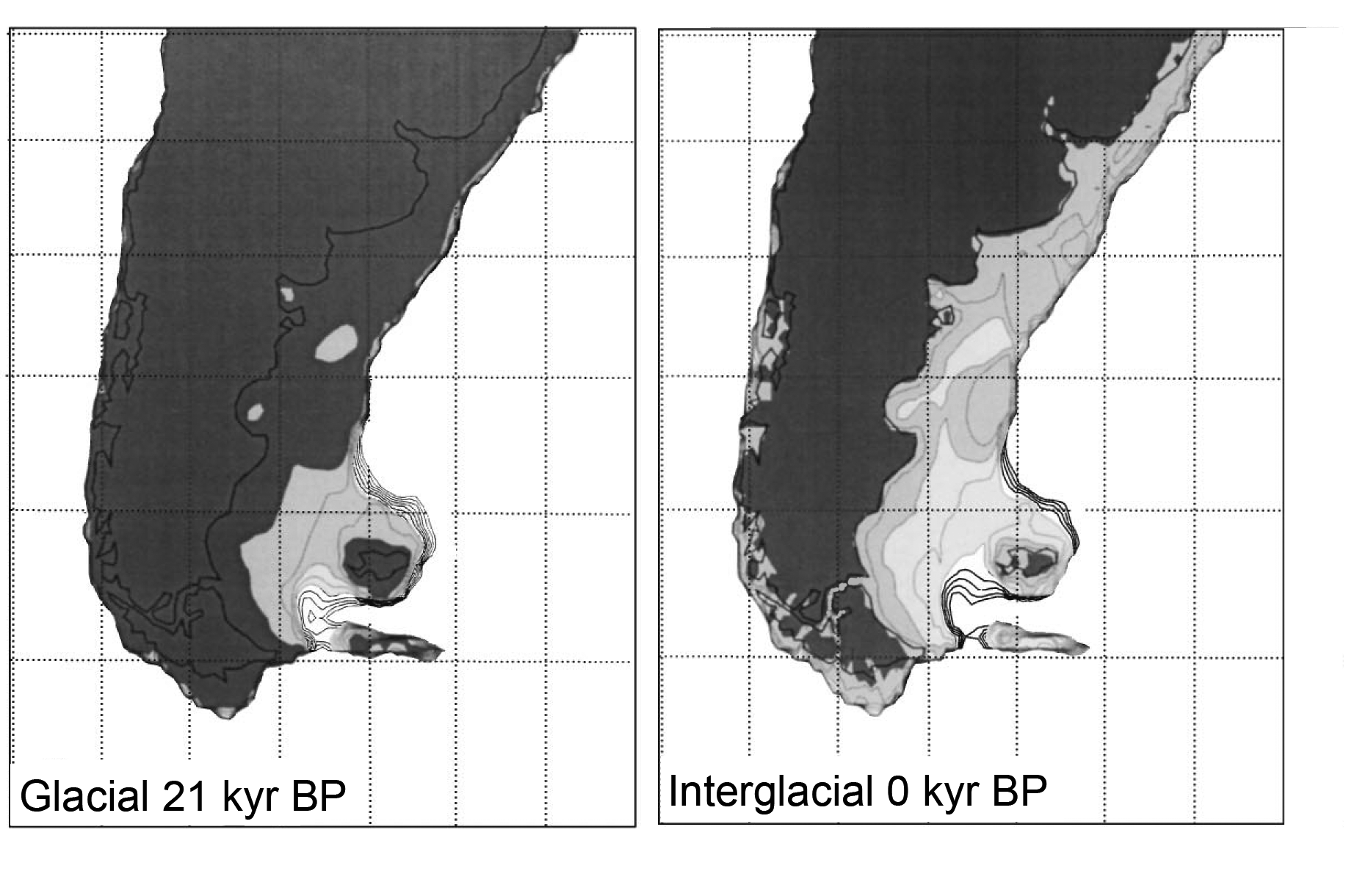

Supplement: Additional file 1 — Terrestrial (black) and marine habitats (grey, 50 m bathymetry isolines) available at last glacial maximum (approximately 21 KY BP, left panel) and at the present interglacial (right panel). During last glacial maximum the sea level was considerably lower thus bringing shallow water habitats of the Patagonian Coast and the Falkland Islands in close proximity. Adapted from [49]. [file 1742-9994-5-19-S1.tiff]
