# Supplementary material for: Cryptic speciation in a benthic isopod from Patagonian and Falkland Island waters and the impact of glaciations on its population structure
Source: Front Zool. 2008 Dec 19;5:19. doi: 10.1186/1742-9994-5-19 (PMC2644686; doi:10.1186/1742-9994-5-19)
Supplement: Additional file 2 — Allele frequencies at seven microsatellite loci for three Serolis paradoxa populations (PA = Punta Arenas, AO = Atlantic opening of the Strait of Magellan, FI = Falkland Islands). Private alleles are shown in bold. [file 1742-9994-5-19-S2.pdf]

Supplement File 2: Allele frequencies at seven microsatellite loci for three *Serolis paradoxa* populations (PA=Punta Arenas, AO=Atlantic opening of the Strait of Magellan, FI=Falkland Islands).

		Location		
**Locus**	**Allele**	**PA**	**AO**	**FI**
**Spa04**				
	126	0.219	-	-
	128	-	-	0.063
	130	0.203	0.157	0.854
	132	0.063	0.057	0.042
	134	0.484	0.714	-
	136	-	0.057	0.042
	138	-	0.014	-
	140	0.031	-	-
**Spa12**				
	186	-	-	0.021
	188	-	0.015	0.958
	190	0.014	0.030	0.021
	192	0.071	-	-
	194	0.829	0.939	-
	196	0.043	0.015	-
	198	0.029	-	-
	202	0.014	-	-
**Spa34**				
	145	0.043	0.076	-
	147	-	-	0.042
	149	0.043	-	-
	151	0.014	-	-
	153	0.014	-	0.021
	155	-	-	0.021
	157	0.029	0.061	0.063
	159	0.057	0.030	0.292
	161	0.071	0.030	0.104
	163	0.043	0.015	0.042
	165	0.086	0.030	0.042
	167	0.014	0.015	0.083
	169	0.043	-	-
	171	0.157	0.061	0.042
	173	0.186	0.485	-
	175	0.057	0.121	-
	177	0.029	0.061	0.063
	179	0.014	-	0.146
	181	0.029	-	-
	183	-	0.015	0.021
	185	0.043	-	-
	187	0.014	-	0.021
	189	0.014	-	-
**Spa35**				
	227	-	0.017	-
	231	-	0.033	-
	235	0.485	0.467	-
	237	0.074	0.117	-
	239	0.015	0.050	0.118
	241	0.309	0.183	-
	243	0.074	0.100	0.029
	245	0.029	-	0.706

	247	-	0.017	0.088
	249	0.015	-	-
	251	-	-	0.059
	255	-	0.017	-
**Spa39**				
	172	0.071	-	-
	180	0.014	0.018	-
	184	-	0.018	-
	188	0.157	0.232	-
	192	0.043	0.161	0.077
	196	0.114	0.054	-
	200	0.071	0.089	-
	202	-	-	0.231
	204	0.071	0.071	0.154
	206	-	0.054	0.385
	208	0.071	-	-
	210	0.014	-	-
	212	-	0.018	0.077
	216	0.129	0.036	-
	220	0.043	0.107	-
	224	0.057	0.054	-
	228	0.086	0.018	-
	232	0.014	-	-
	234	-	-	0.077
	236	0.014	0.036	-
	240	0.014	0.018	-
	244	0.014	-	-
	256	-	0.018	-
**Spa42**				
	150	-	-	0.044
	154	-	-	0.022
	158	-	-	0.065
	160	-	-	0.109
	162	0.014	0.014	0.065
	164	-	-	0.087
	166	0.057	0.071	0.152
	168	0.086	0.086	0.217
	169	-	-	0.022
	170	0.143	0.143	0.174
	172	0.157	0.143	0.044
	174	0.114	0.214	-
	176	0.114	0.114	-
	178	0.129	0.043	-
	180	0.057	0.071	-
	182	0.014	0.029	-
	184	0.043	-	-
	186	-	0.014	-
	188	0.029	0.014	-
	190	-	0.014	-
	192	0.014	0.029	-
	206	0.029	-	-
**Spa43**				
	176	-	0.014	-
	177	0.257	0.300	0.022
	178	0.743	0.671	0.239
	182	-	0.014	0.109
	183	-	-	0.609
	184	-	-	0.022
