# Supplementary material for: Cryptic speciation in a benthic isopod from Patagonian and Falkland Island waters and the impact of glaciations on its population structure
Source: Front Zool. 2008 Dec 19;5:19. doi: 10.1186/1742-9994-5-19 (PMC2644686; doi:10.1186/1742-9994-5-19)
Supplement: Additional file 3 — Statistical tests for significant heterozygosity (H) excess or deficiency in three populations of Serolis paradoxa (PA, AO, FI) assuming three different mutation models (IAM, TPM, SMM). P-values of the Sign Test and Standardized Differences Test and one-tailed probability for heterozygosity deficiency are based on a 1000 permutations. Significant P-values are printed in bold. The TPM was adjusted to allow for 80% mutations according to a SMM and 20% to an IAM model. [file 1742-9994-5-19-S3.pdf]

Supplement File 3: Statistical tests for significant heterozygosity (H) excess or deficiency in three populations of *Serolis paradoxa* (PA, AO, FI) assuming three different mutation models (IAM, TPM, SMM). P-values of the Sign Test and Standardized Differences Test and one-tailed probability for heterozygosity deficiency are based on a 1000 permutations. Significant P-values are printed in bold. The TPM was adjusted to allow for 80% mutations according to a SMM and 20% to an IAM model.

		**Sign Test**				**Wilcoxon Test**
Population	Model	Expected no of loci with H excess	Observed no. of loci with H excess	observed no of loci with H deficiency	P	P (one tailed for H deficiency)
**PA**	IAM	4.00	6	1	0.1212	0.9453
	TPM	4.04	4	3	0.6306	0.7109
	SMM	4.03	3	4	0.3360	0.2891
**AO**	IAM	4.17	3	4	0.2979	0.2343
	TPM	4.10	1	6	**0.0226**	**0.0117**
	SMM	4.09	0	7	**0.0021**	**0.0039**
**FI**	IAM	4.06	4	3	0.6244	0.3438
	TPM	4.14	2	5	0.1049	0.1484
	SMM	4.16	2	5	0.1024	**0.0195**
