# Supplementary material for: Cryptic speciation in a benthic isopod from Patagonian and Falkland Island waters and the impact of glaciations on its population structure
Source: Front Zool. 2008 Dec 19;5:19. doi: 10.1186/1742-9994-5-19 (PMC2644686; doi:10.1186/1742-9994-5-19)
Supplement: Additional file 4 — Mismatch analysis based on the 16S rDNA data sets. Confidence intervals (CI give the 5% and 95% values, respectively for the parameters estimated by 50,000 bootstrap replicates. [file 1742-9994-5-19-S4.pdf]

Supplement File 4: Mismatch analysis based on the 16S rDNA data sets. Confidence intervals (CI give the 5% and 95% values, respectively for the parameters estimated by 50,000 bootstrap replicates.

Parameter	Magellan Strait (PA)	Atlantic Opening (AO)	Falkland Islands (FI)
Tau	0.830	3.000	3.000
CI (Tau)	0.301-1.559	0.387-3.500	0.359-3.500
Theta 0	0.005	0.0	0.0
CI (Theta 0)	0.000-0.109	0.0-0004	0.0-0.004
Theta 1	inf.	0.513	0.370
CI (Theta 1)	6.393-inf.	0.0-inf.	0.0-inf.
SSD	0.019 (P=0.108)	0.001 (P=0.708)	0.004 (P=0.471)
Raggedness	0.166 (P=0.104)	0.211 (P=0.668)	0.298 (P=0.601)
